# Supplementary material for: Monitoring breast cancer response to neoadjuvant chemotherapy with ultrasound signal statistics and integrated backscatter
Source: PLoS One. 2019 Mar 14;14(3):e0213749. doi: 10.1371/journal.pone.0213749 (PMC6417657; doi:10.1371/journal.pone.0213749)
Supplement: S1 Table — (PDF) [file pone.0213749.s001.pdf]

Tumor no.	Response type	IBSC relative change [%]					ENS relative change [%]					Size relative change [%]				
		course no. 1	course no. 2	course no. 3	course no. 4	course no. 5	course no. 1	course no. 2	course no. 3	course no. 4	course no. 5	course no. 1	course no. 2	course no. 3	course no. 4	course no. 5
1	non-resp.	206	17	60	57	4	70	35	17	32	104	35	-15	-40	-35	-35
2	non-resp.	-34	-10	-50	-	-	-7	-13	7	-	-	8	-8	8	-	-
3	non-resp.	4	40	-13	-39	-	-1	8	-36	-50	-	-4	-22	-26	-30	-
4	non-resp.	76	4	-14	57	-47	6	5	-5	2	3	20	-20	-7	0	13
5	non-resp.	-74	-55	-33	14	-43	-5	-2	0	7	19	-38	-19	-19	-23	-19
6	resp.	-22	-20	-23	-46	-15	-58	-62	-63	-58	-71	0	-10	-30	-40	-60
7	resp.	41	12	122	25	34	-39	1	-2	-54	-57	59	0	-4	-52	-70
8	resp.	-15	36	121	180	232	-67	-43	-79	-71	-56	7	2	-24	-59	-63
9	resp.	-28	-41	-11	-60	54	-44	-43	-37	-60	-86	-41	-26	-22	-41	-44
10	resp.	-43	267	170	124	95	-19	-24	-28	-21	-34	32	12	-12	-36	-52
11	resp.	-1	23	16	91	-	-37	24	-32	-35	-	-32	-45	-58	-55	-
12	resp.	-23	0	78	136	-	-13	-10	-11	-95	-	4	-13	-8	-38	-
13	resp.	3	8	28	47	284	-23	-17	-92	-99	-119	-41	-41	-19	-19	-41
14	resp.	-15	36	121	180	232	13	-81	-92	-98	-126	-28	-28	-38	-41	-45
15	resp.	-15	76	36	121	-	-15	-76	-36	-121	-	-17	0	17	0	-
16	resp.	81	-20	-34	56	82	20	58	26	13	-21	-14	-33	-43	-48	-62
17	resp.	-15	-60	-6	120	137	24	-65	-100	-84	-70	-10	-30	-20	-20	-20
18	resp.	224	273	-	-	-	-89	-97	-	-	-	20	-20	-	-	-
19	resp.	35	90	137	279	349	86	12	-18	-36	-33	-9	0	-14	-27	-50
20	resp.	32	-5	37	266	-	2	-5	-8	-116	-	23	-15	-23	-46	-
21	resp.	12	48	87	115	-	-30	-30	-53	-62	-	17	17	-25	-42	-
22	resp.	35	70	95	58	-	3	-6	-17	-23	-	6	3	0	-6	-
23	resp.	44	73	56	160	-	3	-12	-55	-79	-	-20	-35	-35	-40	-
24	resp.	114	207	233	147	-	-2	-25	-35	-114	-	0	-9	-18	-18	-
